# Bacterial Community of Galchi-Baechu Kimchi Based on Culture-Dependent and - Independent Investigation and Selection of Starter Candidates

**DOI:** 10.4014/jmb.2112.12003

**Published:** 2021-12-29

**Authors:** Tao Kim, Sojeong Heo, Hong-Eun Na, Gawon Lee, Jong-Hoon Kim, Mi-Sun Kwak, Moon-Hee Sung, Do-Won Jeong

**Affiliations:** 1 Department of Food and Nutrition, Dongduk Women’s University, Seoul 02748, Republic of Korea; 2 Department of Bio and Fermentation Convergence Technology, Kookmin University, Seoul 02707, Republic of Korea; 3 KookminBio Corporation, Seoul 02826, Republic of Korea

**Keywords:** Galchi-baechu kimchi, bacterial community, culture dependent, culture independent, *Bacillus*, starter

## Abstract

In this study, the bacterial community of galchi-baechu kimchi was determined using culture-based and culture-independent techniques (next generation sequencing:NGS), and showed discrepancies between results. *Weissella koreensis* and *Pediococcus inopinatus* were the dominant species according to the NGS results, while *Bacillus* species and *P. inopinatus* were dominant in the culture-dependent analysis. To identify safe starter candidates, sixty-five *Bacillus* strains isolated from galchi-baechu kimchi using culture-dependent methods were evaluated for their antibiotic resistance, presence of toxin genes, and hemolytic activity. Strains were then assessed for salt tolerance and protease and lipase activity. As a result, four strains-*B. safensis* GN5_10, *B. subtilis* GN5_19, *B. velezensis* GN5_25, and *B. velezensis* GT8-were selected as safe starter candidates for use in fermented foods.

## Introduction

Kimchi is a traditional Korean fermented food used in a variety of side dishes, as an ingredient for soups, or with rice and noodles. There are over 160 different types of kimchi, varying by the main ingredient, minor ingredients, region, season, and in other ways [1]. The names of different kimchi varieties depend on the main ingredient and the representative type of kimchi is called baechu-kimchi, which is made mostly with baechu (cabbage, *Brassica rapa*) along with various minor ingredients. Basic minor ingredients include radish, carrot, leek, garlic, onion, mustard, and seasonings. Ingredients such as fish are also added into kimchi in some regions. The added fish type varies by area: junchi (*Ilisha elongata*) in the western central coastal region, and galchi (hairtail; *Trichiurus lepturus*) and jogi (croaker; *Micropogonias undulatus*) in the southern coastal region.

Lactic acid bacteria (LAB) of the genera *Leuconostoc* (*Leu.*), *Lactobacillus* (*Lb.*), *Weissella*, *Lactococcus*, and *Pediococcus* are the dominant bacteria during kimchi fermentation [2]. In baechu-kimchi fermentation, the LAB community starts with heterofermentative LAB such as *Leuconostoc* spp., followed by *Weissella* spp., and then homofermentative *Lactobacillus* spp. dominate [2-5]. Although this change in the microbial structure is well known, most studies have focused on baechu-kimchi, the most representative variety, and the microbial communities of regional varieties of kimchi have rarely been described. Recently, we isolated *Bacillus* strains from a regional variety, galchi-baechu kimchi [6, 7]. In these studies, we assumed that the microbial structure of galchi-baechu kimchi might be distinct from that of the representative baechu-kimchi. Moreover, we are unaware of studies designed to identify the predominant species in galchi-baechu kimchi. Therefore, we investigated the bacterial community of galchi-baechu kimchi using culture-independent and -dependent analyses. Additionally, starter candidates were selected from among the isolated strains following safety and functional assessments.

## Materials and Methods

### Kimchi and Bacterial Strain Isolation

Homemade galchi-baechu kimchi samples were prepared in the city of Pohang in Gyeongsangbuk-do Province; these samples were fermented for 2 months at 10°C. Samples were ground, homogenized, or both, with an equal amount of sterilized water, then filtered through sterilized cheesecloth. For microbial counting, the filtrates were spread on four types of agar medium: Plate Count Agar (PCA; BD, USA), Tryptic Soy Agar (TSA; BD), Nutrient Agar (NA; BD), and Lactobacilli De Man, Rogosa and Sharpe Agar (MRS; BD); all media were incubated at 30°C for 24 h.

### Determination of pH and NaCl Concentration

The filtrates were analyzed for pH and NaCl concentration. The pH was measured using a pH meter (MP220, USA). NaCl concentration was measured using silver nitrate according to the Mohr method [8]. All experiments were conducted in triplicate.

### Pyrosequencing of Culture-Independent Samples

To perform metagenomic analysis of the bacterial community, a DNeasy PowerSoil Kit (Qiagen, Germany) was used to prepare DNA from filtrates of kimchi. DNA quantity and quality were determined using PicoGreen and a NanoDrop spectrometer (Thermo Fisher Scientific, USA). The V3–V4 hypervariable regions of the bacterial 16S rRNA genes from the isolated genomic DNA were amplified using the primer set 5′-TCG TCG GCA GCG TCA GAT GTG TAT AAG AGA CAG CCT ACG GGN GGC WGC AG-3′ and 5′-GTC TCG TGG GCT CGG AGA TGT GTA TAA GAG ACA GGA CTA CHV GGG TAT CTA ATC C-3′, and a MyCycler Thermal Cycler (Bio-Rad, USA). PCR mixtures contained 30 ng of genomic DNA, 50 pM each primer, and Han-*Taq* polymerase (Genenmed, Korea). PCR was performed as follows: initial denaturation at 94°C for 90 s; 30 cycles of denaturation at 94°C for 45 s, annealing at 55°C for 45 s, extension at 72°C for 45 s; and final extension at 72°C for 5 min.

Each primer was concatenated to an eight-base, sample-specific barcode sequence and a common linker sequence [TC (forward primer) and CA (reverse primer)] at the 5′- terminus [9]. Pyrosequencing was performed by Macrogen (Korea) using the MiSeq platform (Illumina, USA) in accordance with the manufacturer’s instructions. To assess richness estimators of bacterial species, diversity indices, and rarefaction curves, we applied the pyrosequencing pipeline of the Ribosomal RNA Database Project [10].

### Analysis of Culture-Dependent Bacterial Community Using 16S rRNA Gene Sequence Analysis

Bacteria were isolated using TSA, NA, and MRS. Filtrates were spread on agar media after appropriate dilution. All media were incubated at 30°C until distinguishable colonies appeared; >20 colony types were collected from each plate according to differences in morphology and growth characteristics. The colonies were purified by successive transfers to plates containing the same type of agar medium used for isolation.

Genomic DNAs of isolates were extracted using a DNeasy Blood & Tissue Kit (Qiagen). Amplification of 16S rRNA genes was performed using eubacterial universal primers 27F (5′-AGA GTT TGA TCC TGG CTC AG-3′) and 1492R (5′-GGT TAC CTT GTT ACG ACT T-3′) [11] with a T3000 Thermocycler (Biometra, Germany). The PCR mixture comprised template DNA (10 ng), 0.5 mM each primer, 1 U of *Taq* polymerase (Inclone Biotech, Korea), 10 mM dNTPs, and 2.5 mM MgCl_2_. Samples were heated for 5 min at 95°C and then amplified using 30 cycles of 1 min at 95°C, 1 min at 58°C, and 1 min at 72°C. The PCR products were purified and sequenced using a custom service provided by Macrogen. We used a web-hosted BLASTn algorithm to query the National Center for Biotechnology Information database for 16S rRNA gene sequences (http://blast.ncbi.nlm.nih.gov). The adenylate kinase (*adk*) gene was used to precisely confirm the taxonomic identity of *Bacillus* strains [12].

### Safety Assessment of Isolates

**Antibiotic Susceptibility**

Antibiotic susceptibility was determined by the broth microdilution method using eight antibiotics: ampicillin, chloramphenicol, clindamycin, erythromycin, gentamicin, streptomycin, tetracycline, and vancomycin. Antibiotics were prepared with serial twofold working dilutions in deionized water and the final concentration of each antibiotic ranged between 0.5 and 32 mg/l [13]. Cultured bacterial strains in Mueller-Hinton broth (MH broth; BD) were matched to a 0.5 McFarland turbidity standard (bioMérieux, France). Each suspension was further diluted 1:100 in cation-adjusted MH broth to achieve an appropriate inoculum concentration. The final inoculum density was 5 × 10^5^ colony-forming units (CFU)/ml, 200 μl of which was added to each well of a 96-microwell plate. The minimum inhibitory concentration (MIC) of each antibiotic was recorded as the lowest concentration where no growth was observed in the wells after incubation at 37°C for 24 h. MIC results were confirmed by at least three independently performed tests. All the experiments were conducted at least three times on separate days. Microbiological breakpoints are set by studying the distribution of MICs of the chosen antimicrobials in bacterial populations belonging to a single taxonomic unit (species or genus). Strains with an MIC higher than the breakpoint are considered resistant [13].

**Presence of Enterotoxin Genes**

Ten enterotoxin genes were amplified from genomic DNA using specific primer sets (Table 1) [14, 15]. The PCR reactions were performed using Inclone *Taq* polymerase in accordance with the manufacturer’s recommended methods. The amplicons were checked on 1.2% agarose gels. *Bacillus cereus* KCCM 11341 was used as a positive control.

**Hemolysis**

Hemolytic activity was determined by halo formation around colonies on TSA supplemented with 5% (v/v) rabbit blood (MBcell, Korea) or 5% (v/v) sheep blood (MBcell) for α- and β-hemolytic activity tests, respectively. α-Hemolytic activity was determined by incubation at 37°C for 24 h, and β-hemolytic activity was determined by cold shock at 4°C for 24 h after incubation at 37°C for 24 h. Clinically isolated *Staphylococcus aureus* USA300-P23 and RN4220 were used as positive and negative controls, respectively [34]. The experiments were performed in triplicate.

**Technological Assessments**

Protease activity was determined on TSA containing 2% (w/v) skim milk (BD). Lipase activity was tested on tributyrin agar (Sigma-Aldrich, USA) containing 1% (v/v) tributyrin. The tributyrin-supplemented medium was emulsified by sonication before autoclaving. Acid production was determined on TSA supplemented with 0.7%(w/v) CaCO3 (Sigma-Aldrich). Strains were cultured on appropriate substrate-supplemented agar medium and incubated at 37°C for 24 h. The relative size of the zone of clearing around the colony was used as an indicator of enzyme activity. The effect of NaCl on each activity was determined by the addition of NaCl to each medium, up to a final concentration of 6% (w/v).

Salt tolerance of *Bacillus* strains was determined by examining growth on TSA supplemented with up to 15%(w/v) NaCl final concentration. Growth on 5, 10, and 15% NaCl was determined after 2-, 3-, and 4-day incubations, respectively.

## Results

### Kimchi Conditions and Viable Cell Counts

Galchi-baechu kimchi samples had an average NaCl concentration of 2.13% and average pH of 4.07. Generally, the pH of baechu-kimchi decreases to approximately 4.0 during fermentation, and the NaCl concentration is around 2–5% (w/w) NaCl [9-11]. Therefore, the kimchi samples used in this work were similar in pH and NaCl concentration to ordinary commercially sold kimchi.

The average concentrations of the bacterial populations of galchi-baechu kimchi determined using different media in this study ranged from 1.5 × 10^6^ CFU/g to 2.1×10^8^ CFU/g (Table 2). There were more viable cells on the MRS medium than on the other media.

### Bacterial Community Based on Culture-Independent Analysis

Pyrosequencing analyses of bacterial DNA isolated from galchi-baechu kimchi determined 217,428 sequences of sufficient quality. The sequence coverage was 1.00, which provided sufficient statistical power to conduct analyses of the bacterial community. The numbers of operational taxonomic units and the Chao1 (species richness) and Shannon indices (species diversity) are shown in Table 3. The Chao1 and Shannon indices were 28 and 1.58, respectively.

**Fig. 1:**
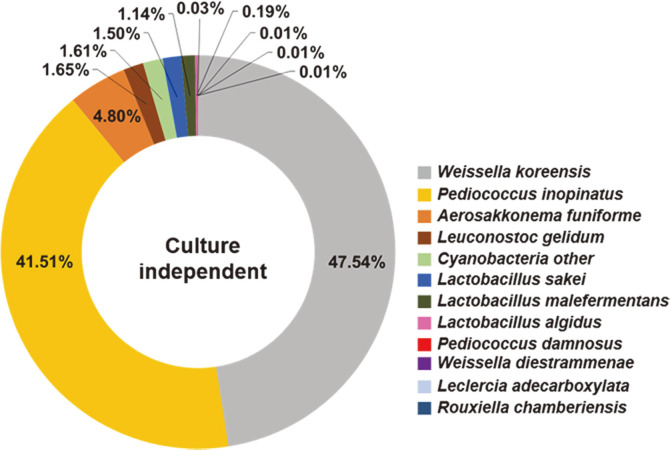
Bacterial species composition of kimchi. Data portray levels of species identified from the V3/V4 regions of 16S rRNA gene sequences by pyrosequencing. Regions >200 bp were classified using CD-HIT-OUT (97% confidence threshold).

The phylogenetic classification of the bacterial community is summarized in Fig. 1. Members of the phyla Firmicutes (93.57%), Cyanobacteria (6.41%), and Proteobacteria (0.02%) were identified. Among the Firmicutes, *Weissella* (47.55%) and *Pediococcus* (41.54%) were the predominant genera. The predominant species were *W. koreensis* (47.54%), *P. inopinatus* (41.51%), and *Aerosakkonema funiforme* (4.80%).

### Bacterial Community Based on Culture-Dependent Analysis

Using three different agar media, we isolated 102 strains from galchi-baechu kimchi and those strains were identified by 16S rDNA gene analysis (Table 4). *Bacillus* species were confirmed using the adk gene because of the high similarity with *Bacillus* 16S rRNA genes [12]. The isolates were determined to belong to 10 species in the family Bacillaceae: five species in the genus *Bacillus*; three LAB species—*Lb. curvatus*, *Leu. mesenteroides*, and *Pediococcus inopinatus*; and *Priestia flexa* and *Terribacillus aidingensis*. *Bacillus* spp. accounted for 63.72% (65/ 102) of the total isolates. Notably, *B. subtilis* (25.49% of the total isolates) and *B. velezensis* (21.57% of the total isolates) were the most populous species among the *Bacillus* spp. *Pediococcus inopinatus* was the predominant species among the LAB and the third dominant species (19.61% of the total isolates) overall.

Interestingly, different bacterial species were isolated dependent upon the medium used; more *Bacillus* than LAB were detected on TSA and NA media; only LAB were isolated using MRS.

### Safety Properties of *Bacillus* Species

To use a bacterial starter in the food industry, it must be safe. The European Union introduced the Qualified Presumption of Safety (QPS) system to check the safety of microorganisms for food and feed [16]. The European Food Safety Authority (EFSA) produced QPS lists every year until 2013 and every 3 years thereafter. As of August 2021, 16 *Bacillus* species are on the QPS list [17]. To consider a *Bacillus* species safe, the QPS guidelines require a lack of acquired antibiotic resistance genes and the absence of toxigenic activity. Additionally, *B. velezensis* and *B. circulans* are required to show absence of toxigenic potential and absence of aminoglycoside production ability, for production purposes only and absence of cytotoxic activity, respectively [17]. We checked the antibiotic susceptibility, presence of enterotoxin genes, and hemolysis, including resistance to aminoglycosides for the *Bacillus* strains isolated in this study based on the QPS guidelines.

**Fig. 2:**
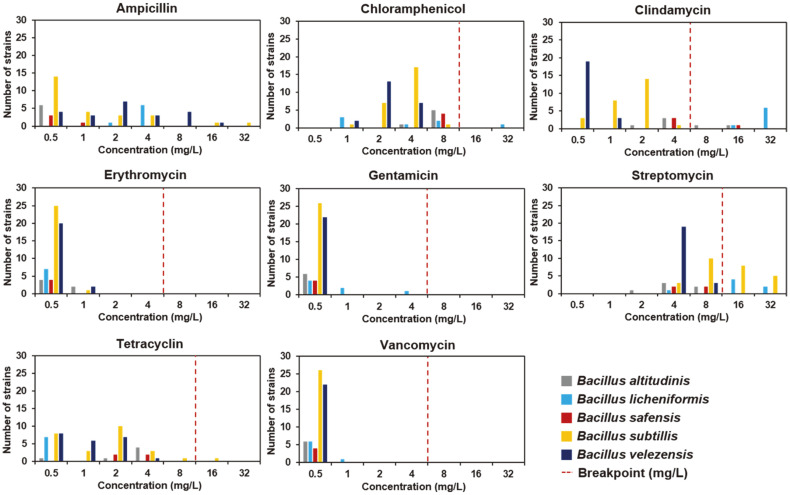
Minimum inhibitory concentration distributions for eight antibiotics and 65 *Bacillus* strains isolated from kimchi, determined by the broth microdilution method. Breakpoints are the values for *Bacillus* suggested by the European Food Standards Authority [13]. The breakpoint for ampicillin has not been defined.

To test the antibiotic resistance, we selected eight antibiotics (ampicillin, chloramphenicol, clindamycin, erythromycin, gentamicin, streptomycin, tetracycline and vancomycin) [13]; the MICs of the tested antibiotics toward the 65 *Bacillus* isolates are summarized in Fig. 2; all 65 *Bacillus* strains were sensitive to erythromycin, gentamicin, and vancomycin based on EFSA criteria [13]. One *B. licheniformis* strain and one *B. subtilis* strain showed a high MIC compared with the breakpoints for chloramphenicol and tetracycline, respectively. The MIC of clindamycin ranged from 0.5 to 32 mg/l, and the MICs were higher than the breakpoint for 10 isolates (two strains of *B. altitudinis*, seven *B. licheniformis*, and one *B. safensis*) (Fig. 2). In a previous study, we suggested that clindamycin resistance might be an intrinsic feature of *B. licheniformis* through gene mutations [18]. Nineteen strains showed MIC values of streptomycin higher than the breakpoint.

In accordance with the QPS guidelines [17], we also checked the presence of toxigenic determinants in *Bacillus* species, based on toxin genes of pathogenic *B. cereus* (Table 1). *B. cereus* has three hemolytic enterotoxin genes (*hblA*, *hblC*, and *hblD*), three non-hemolytic enterotoxin genes (*nheA*, *nheB*, and *nheC*), a cytotoxin K gene (*cytK*), enterotoxin genes (*bceT*, *entFM*), and an emetic toxin gene (*ces*). Fortunately, those toxin genes were not identified in any of our *Bacillus* isolates. Furthermore, none of the strains exhibited hemolysis on rabbit or sheep blood media.

### Technological Properties of *Bacillus* Species

To find useful strains for preparing fermented foods, protease and lipase activities are generally determined. These enzymes contribute to the sensory properties of food through protein and lipid degradation. The protease and lipase activities of the *Bacillus* species isolated in this study were strain-specific, and the activities decreased or disappeared as the NaCl concentration was increased (Table 5). Thirteen strains retained protease activity at an NaCl concentration of 6%, while most of the strains showed protease activity at 0.5% NaCl. Twenty-six strains exhibited lipase activity in conditions without added NaCl, and nine strains showed lipase activity at 2% NaCl.

Although the requirements of a starter culture have not been clearly determined, most Korean fermented foods are made in high-salt conditions [19]. Therefore, we assumed that salt-tolerance is also an important requirement for a starter candidate. We checked the salt tolerance of our strains. All strains grew in up to 5% NaCl; only one strain grew at 15% NaCl.

On the basis of our analyses, we identified safe starter candidates showing efficient technological properties (high-salt-tolerance, high proteolysis, and high lipolysis; Table 6); strains with MIC values above the breakpoint for any of the eight antibiotics were excluded. Four starter candidates met the criteria: *B. safensis* GN5_10, *B. subtilis* GN5_19, *B. velezensis* GN5_25, and *B. velezensis* GT8.

## Discussion

Culture-independent methods to determine the bacteria community were not affected by media, because they are used to analyze DNA extracted from samples. However, it is difficult to distinguish between viable and dead bacteria, and it is not easy to secure microorganisms. Culture-dependent analyses compensate for disadvantages of culture-independent analysis, but there is prejudice in the media. In the current experiment, culture-independent and culture-dependent analyses were applied to confirm differences in bacterial community in galchi-baechu kimchi (Fig. 1, Table 4). Pyrosequencing results showed a more diverse bacterial community than culture-dependent analysis. The only shared species between the two methods was *P. inopinatus*. Interestingly, the dominantly detected *Bacillus* species from TSA and NA agar were not detected by pyrosequencing. *W. koreensis* was not isolated by culture-dependent analysis, although *W. koreensis* was found to be dominant by pyrosequencing.

*P. inopinatus* belongs to the family Lactobacillaceae and is frequently isolated from various types of kimchi [20-22]. Although *P. inopinatus* contributes to acidification in foods [23], research on the correlations between *P. inopinatus* and Kimchi is insufficient.

*Weissella koreensis* belongs to the family Leuconostocaceae and is the dominant species found in kimchi [24]. In the current study, *W. koreensis* was dominant based on pyrosequencing, but was not isolated using three media, including MRS, which is an appropriate medium for *W. koreensis*. Yu *et al*. reported that the optimum pH and temperature for *W. koreensis* are 6.5 and 30°C, respectively, and *W. koreensis* did not grow at approximately pH 4.0 [25]. The pH of the current galchi-baechu kimchi sample was 4.07. Therefore, we assume that *W. koreensis* was not viable in our galchi-baechu kimchi and the pyrosequencing results detected DNA of dead bacteria remaining in the galchi-baechu kimchi.

*Bacillus* species were previously detected in kimchi by culture-independent methods or using TSA, not MRS [9, 26, 27]. In a previous study, we showed that *Bacillus* formed endospores during kimchi fermentation [9]. In the current study, *Bacillus* species were dominant among the bacteria detected by the culture-dependent method using TSA and NA media. Meanwhile, those strains were not identified by pyrosequencing. Therefore, we assume that *Bacillus* species were present as endospores in the kimchi.

*Bacillus* species were not active in the galchi-baechu kimchi used in this study, but *Bacillus* species have several advantages as starter candidates for several fermented foods such as fermented soybean products such as meju and doenjang [28, 29]. *Bacillus* produce proteases and lipases [30-32]. These enzyme activities contribute to the improvement of quality and sensory properties of fermented foods [33-35]. *Bacillus* species also grow on media supplemented with NaCl [19]. These properties are big advantages for industrial food strains, and selected starter candidates in the current study might have contributed the sensory properties in fermented food via volatile and taste compounds production, which are produced from macromolecules by enzyme activity.

The present study reveals the advantages and limitations of culture-independent and culture-dependent analysis of microbial communities. The former is a powerful method to evaluate microbial diversity, but it does not distinguish between viable and dead cells. Although the latter method isolates viable cells, it is highly dependent on the type of medium used and does not sufficiently assess the whole microbial community. Complementary results using the two methods may more fully explain bacterial communities.

## Figures and Tables

**Table 1:** Primer sets for the detection of toxin factors in 65 *Bacillus* species.

Target gene	Oligonucleotide sequence (5'-3')		Size (bp)	Reference

	Forward	Reverse		
Hemolytic enterotoxin genes				
*hblA*	AAGCAATGGAATACAATGGG	AGAATCTAAATCATGCCACTGC	1,154	[14]
*hblC*	GATACYAATGTGGCAACTGC	TTGAGACTGCTCGYTAGTTG	740	[14]
*hblD*	ACCGGTAACACTATTCATGC	GAGTCCATATGCTTAGATGC	582	[14]
Non-hemolytic enterotoxin genes				
*nheA*	TACGCTAAGGAGGGGCA	GTTTTTATTGCTTCATCGGCT	499	[14]
*nheB*	CTATCAGCACTTATGGCAG	ACTCCTAGCGGTGTTCC	769	[14]
*nheC*	CGGTAGTGATTGCTGGG	CAGCATTCGTACTTGCCAA	581	[14]
Cytotoxin K gene				
*cytK*	ATCGGKCAAAATGCAAAAACACAT	ACCCAGTTWSCAGTTCCGAATGT	515	[15]
Enterotoxin genes				
*bceT*	CGTATCGGTCGTTCACTCGG	TTTCTTTCCCGCTTGCCTTT	924	[14]
*entFM*	AAAGAAATTAATGGACAAACTCAAACTCA	GTATGTAGCTGGGCCTGTACGT	596	[15]
Emetic toxin gene				
*ces*	TTGTTGGAATTGTCGCAGAG	GTAAGCGAACCTGTCTGTAACAACA	405	[15]

**Table 2:** Viable bacterial counts on different media.

Medium	Cell count (CFU/g)
PCA	1.8×10^6^ ± 1.1×10^6^
TSA	1.5×10^6^ ± 3.2×10^5^
NA	1.3×10^6^ ± 2.5×10^5^
MRS	2.1×10^8^ ± 1.4×10^7^

PCA, Plate Count Agar; TSA, Tryptic Soy Agar; NA, Nutrient Agar; MRS, Lactobacilli De Man, Rogosa and Sharpe Agar.

**Table 3:** Microbial diversity indices of kimchi.

Sample name	High-quality reads	OTUs	Chao1	Shannon	Good’s coverage
Kimchi	217,428	26	28	1.58	1.00

OTUs, operational taxonomic units.

**Table 4:** Numbers of bacterial species isolated from galchi-baechu kimchi using different media.

Species	TSA	NA	MRS	Total
*Bacillus altitudinis*	3	3		6
*Bacillus safensis*		4		4
*Bacillus subtilis*	8	18		26
*Bacillus licheniformis*	7			7
*Bacillus velezensis*	7	15		22
*Priestia flexa*		1		1
*Lactobacillus curvatus*			2	2
*Leuconostoc mesenteroides*	3	2	8	13
*Pediococcus inopinatus*			20	20
*Terribacillus aidingensis*	1			1
Total	29	43	30	102

TSA, Tryptic Soy Agar; NA, Nutrient Agar; MRS, Lactobacilli De Man, Rogosa and Sharpe Agar.

**Table 5:** Technological properties of the 65 *Bacillus* isolates.

Species	*n*	NaCl tolerance			Protease activity			Lipase activity	
		
		5%	10%	15%	0.5%^a^	3%	6%	0%	2%
*Bacillus altitudinis*	6	6	5	-	5	2	-	3	1
*Bacillus safensis*	4	4	4	-	4	1	-	4	3
*Bacillus subtilis*	26	26	21	1	25	10	3	8	1
*Bacillus velezensis*	22	22	20	-	22	18	9	9	3
*Bacillus licheniformis*	7	7	6	1	7	1	1	2	1
Total	65	65 (100)	54 (83)	2 (3.1)	64 (98.5)	32 (49.2)	13 (20.0)	26 (40.0)	9 (13.8)

^a^The NaCl concentration in TSA is 0.5% (w/v), and others indicate the final concentration of NaCl in the media.

Numbers in parentheses are percentages.

**Table 6:** Selected starter candidates from among the 65 *Bacillus* strains.

Species	Strain	Salt tolerance			Protease activity^a^			Lipase activity^b^	
		
		5%	10%	15%	0.5%^c^	3%	6%	0%	2%
*Bacillus safensis*	GN5_10	G	G	NG	+	-	-	+	w
*Bacillus subtilis*	GN5_19	G	G	G	++	-	-	w	-
*Bacillus velezensis*	GN5_25	G	G	NG	++++	+++	++	-	-
*Bacillus velezensis*	GT8	G	G	NG	+++	+++	+	w	w

Abbreviations: G, growth; NG, no growth.

^a^Diameter of clear zone: -, 0 mm; +, ~5 mm; ++, 5–10 mm; +++, 10–15 mm; ++++ >15 mm.

^b^Diameter of clear zone: -, 0 mm; w, ~4 mm; +, >4 mm.

^c^The NaCl concentration in TSA is 0.5% (w/v), and others indicate the final concentration of NaCl in the media.
